# The microbiota of water buffalo milk during mastitis

**DOI:** 10.1371/journal.pone.0184710

**Published:** 2017-09-19

**Authors:** Carlotta Catozzi, Armand Sanchez Bonastre, Olga Francino, Cristina Lecchi, Esterina De Carlo, Domenico Vecchio, Alessandra Martucciello, Pasquale Fraulo, Valerio Bronzo, Anna Cuscó, Sara D’Andreano, Fabrizio Ceciliani

**Affiliations:** 1 Dipartimento di Medicina Veterinaria, Università degli Studi di Milano, Via Celoria 10, Milano, Italy; 2 Molecular Genetics Veterinary Service (SVGM), Veterinary School, Universitat Autònoma de Barcelona, Bellaterra, Barcelona, Spain; 3 Istituto Zooprofilattico Sperimentale del Mezzogiorno, National Reference Centre for Hygiene and Technologies of Water Buffalo Farming and Productions, Via delle Calabrie, Salerno, Italy; 4 Vetgenomics. Ed Eureka. PRUAB. Campus UAB, Bellaterra, Barcelona, Spain; Gaziosmanpasa Universitesi, TURKEY

## Abstract

The aim of this study was to define the microbiota of water buffalo milk during sub-clinical and clinical mastitis, as compared to healthy status, by using high-throughput sequencing of the 16S rRNA gene. A total of 137 quarter samples were included in the experimental design: 27 samples derived from healthy, culture negative quarters, with a Somatic Cell Count (SCC) of less than 200,000 cells/ml; 27 samples from quarters with clinical mastitis; 83 samples were collected from quarters with subclinical mastitis, with a SCC number greater of 200,000 cells/ml and/or culture positive for udder pathogens, without clinical signs of mastitis. Bacterial DNA was purified and the 16S rRNA genes were individually amplified and sequenced. Significant differences were found in milk samples from healthy quarters and those with sub-clinical and clinical mastitis. The microbiota diversity of milk from healthy quarters was richer as compared to samples with sub-clinical mastitis, whose microbiota diversity was in turn richer as compared to those from clinical mastitis. The core microbiota of water buffalo milk, defined as the asset of microorganisms shared by all healthy milk samples, includes 15 genera, namely *Micrococcus*, *Propionibacterium*, *5-7N15*, *Solibacillus*, *Staphylococcus*, *Aerococcus*, *Facklamia*, *Trichococcus*, *Turicibacter*, *02d06*, *SMB53*, *Clostridium*, *Acinetobacter*, *Psychrobacter* and *Pseudomonas*. Only two genera (*Acinetobacter* and *Pseudomonas*) were present in all the samples from sub-clinical mastitis, and no genus was shared across all in clinical mastitis milk samples. The presence of mastitis was found to be related to the change in the relative abundance of genera, such as *Psychrobacter*, whose relative abundance decreased from 16.26% in the milk samples from healthy quarters to 3.2% in clinical mastitis. Other genera, such as *SMB53* and *Solibacillus*, were decreased as well. Discriminant analysis presents the evidence that the microbial community of healthy and clinical mastitis could be discriminated on the background of their microbiota profiles.

## Introduction

The development of culture-independent techniques by means of high-throughput DNA sequencing has just begun to unravel the impact of large community of micro-organisms, the so called microbiota, on human and animal health [1]. Microbiota establishes mutual relationship with its hosts and the resulting cross-talk extends beyond the balance between tolerance to commensal micro-organisms and developing protection against pathogens [2].

Metagenomic techniques have also revealed how “healthy” microbiota, e.g. the microbial community belonging to healthy individuals, includes potential pathogens. Recent studies on gut microbiota have provided the evidence that the onset of a disease can be the result of a change in the interaction with other microorganisms [3]. A new concept of pathobiome, which can be defined as the microbiota environment integrating also pathogenic agents, is taking shape and has been recently discussed and thoughtfully reviewed [4].

In cows, most of the studies has been carried out on ruminal microbiota [5–9]. A metagenomic approach has also been applied to the relationship between resident microbiomes and the development of reproductive diseases [10–14].

Although the relevance of different bacterial pathogens in mastitis has been known for a long time, the impact of complex community of microbes and their interaction in the development of intramammary infection or mastitis has been only recently, and partially, described [15, 16], and recently reviewed [17]. Milk harbours a wide range of bacteria, many of which cannot be identified by culturing of samples on selective media, leaving therefore as undetected those microorganisms that cannot be cultured. As a consequence, for example, it has been reported that 25% of clinical mastitis caused by bacteria are routinely not detected by means of bacterial culture [18], as confirmed by the finding that bacterial species may be present also in culture-negative samples collected from animals with clinical mastitis [19].

The microbial content of raw and pasteurized milk revealed the presence of a rich and diverse bacterial population [20]. Metagenomic pyrosequencing techniques of bacterial 16S rRNA were applied to investigate milk samples from mastitic and healthy dairy cows, revealing that microbiota were different [15, 19]. Although the concept of milk microbiota as determined by culture independent techniques has been very recently challenged [21], the pyrosequencing of bacterial 16S rRNA could discriminate healthy from sub-clinically and clinically affected quarters [16]. Major pathogens such as *Streptococcus uberis* and *Staphylococcus aureus* were also found in milk from animals with no evidence of inflammatory reaction, suggesting the hypothesis that the development of mastitis can be regarded more as a dysbacteriosis than a primary infection [16].

Water buffaloes provide the most important source of non-cattle milk worldwide (13.2%) [22]. In some countries, such as India, water buffalo milk accounts for the 55% of the total milk produced [23]. The effects of environmental factors and management practices, as well as the stage of lactation, parity and calving season, on physical-content and somatic cell counts (SCC) were recently described [24–26]. Dairy water buffaloes can be affected by mastitis with a frequency only slightly lower as compared to cows [27–29]. Mastitis could therefore have negative impacts on water buffalo dairy economy equal to that on cow dairy farms in term of reducing milk yield, premature culling and cost of therapy [30]. Information about pathogens involved in mastitis occurrence in water buffalo is limited. Culture dependent approaches demonstrated that most frequently isolated bacteria during mastitis are coagulase negative, causing 78% of intramammary infections cases of mastitis [31, 32], *Prototeca* spp. and *Streptococcus pluranimalium* being found occasionally [33, 34].

Culture independent techniques have been applied to the study of mozzarella production, focusing on raw milk, natural whey cultures and curd to the final cheese product [35, 36]. Milk microbiota associated with the health status of water buffalo mammary gland has not been investigated yet.

The aim of the present study is to bridge this gap by providing insights into the microbiota of dairy water buffalo milk related to healthy status by means of high-throughput DNA sequencing of the 16S rRNA genome milk samples from healthy and clinical and sub-clinical mastitis affected quarters in dairy water buffaloes.

## Materials and methods

### Sample collection

One hundred thirty-seven quarter milk samples derived from 88 dairy water buffalo cows belonging to 14 farms, homogeneously distributed in Campania area (Italy), were collected from January to February 2016. The samples were collected after owner permission and the collection methods were consistent with recommendations according to standard procedure by National Mastitis Council [37].

Samples were collected after teat ends have been disinfected with 70% ethylic alcohol and the first strain of milk was discarded. Microbial diversity was analysed after classification of quarter milk as follows: 27 samples were collected from healthy quarters with no clinical signs of mastitis during the present lactation, with two consecutive Somatic Cell Counts (SCC) values lower than 200,000 cells/ml and aerobic culture negative for udder pathogens (H); 27 samples with clinical mastitis (CM) were collected from quarters showing signs of clinical mastitis and aerobic culture positive. Three animals with negative microbiological culture but with very high SCC (> 2400,000 cells/ml) were also included in this group. For 14 samples it was not possible to carry out a reliable SCC due to the very high density of milk. Finally, 83 samples with sub-clinical mastitis (SM) were collected from quarters showing no signs of clinical mastitis but with aerobic culture positive for udder pathogens. Fifteen samples with SCC number greater of 200,000 cells/ml but with negative microbiological culture were also included in this group.

Samples were refrigerated and delivered within 12 hours for SCC and microbiological analysis. Animals that were treated in lactation with antibiotics within the previous 90 days were excluded from the experiment.

### Somatic cells count and microbiological culture

Somatic cells count (SCC) was measured in milk samples using Fossomatic (Foss) apparatus by means of the UNI EN ISO 13366–2: 2007 technique for electronic optical fluorometric counters [38].

Microbiological culture tests were performed for each milk sample using different media: cultures were incubated at 37°C for 24h in aerobic conditions on blood agar (Trypticase Soy Agar with 5% sheep blood), MacConkey agar and Baird Parker Agar; at 37°C for 72h in aerobic conditions on *Prototheca* Isolation Medium (PIM) at 37°C in micro-aerobic conditions on *Mycoplasma* agar. Gram staining, coagulase and oxidase tests were performed on cultures with mastitis pathogens; in particular, *Staphylococcus* spp. culture coagulase detection was carried out using rabbit plasma and then for *Streptococcus* spp. Streptokit-BioMérieux test was employed using Lancefield grouping, in order to identify antigen differences between species.

### DNA extraction

One ml of milk was centrifuged for 10 min at room temperature at 16,100 rcf [16]. The supernatant was discarded and the remaining pellet was resuspended in 250μl of the Power Bead Tube solution of the PowerSoil™ DNA isolation kit (MO BIO), which was used to extract bacterial DNA, according to the manufacturer’s instructions. DNA samples were eluted in 50 μl of C6 solution and stored at -20°C until further processing. Therefore, DNA concentration and purity were analyzed using NanoDrop 2000 Spectrophotometer (Thermo Fisher Scientific, Waltham, Massachusetts, U.S.A) at wavelengths 230, 260 and 280 nm.

### Amplification of the hypervariable V1-V2 region of bacterial 16S rRNA gene by PCR and barcoding

V1-V2 regions of 16S rRNA gene were amplified for each sample [16, 19]. The forward primer was 5’–CCATCTCATCCCTGCGTGTCTCCGACTCAGNNNNNNNNNN*GAT*AGAGTTTGATCCTGGCTCAG-3’, composed of the adapter linker, the Key, the barcode that is different from each sample, the spacer and the conserved bacterial F27 forward primer, respectively. The reverse primer was 5’–CCTCTCTATGGGCAGTCGGTGATTGCTGCCTCCCGTAGGAGT- 3’, composed of the adapter linker and the R338 reverse primer. PCR was carried out following the instructions of Thermo Scientific Phusion Hot Start II High-Fidelity DNA Polymerase Kit; each PCR reaction contained RNAse and DNAse free water, 5x Phusion Buffer HF (5 μl), dNTPs 2mM (2.5 μl), Primer Fw 10mM (1.25 μl), primer Rv 10 mM (1.25 μl) and Phusion High Fidelity Taq polymerase (0.25 μl), and 5 ng of DNA sample in a final volume of 25 μl. The lack of amplification of a negative control for each PCR reaction demonstrated the absence of contamination by reagents that could interfere with the analysis [39]. The thermal profile used for the amplification consisted of an initial denaturation of 30 sec at 98°C, followed by 30 cycles of 30 sec at 98°C, 15 sec at 55°C, 20 sec at 72°C and a final extension of 7 min at 72°C. Each PCR plate included samples derived from each group. Quality and quantity of PCR products were determined using Agilent Bioanalyzer 2100 and Qubit™ fluorometer. All 137 quarter milk samples (27 H, 27 CM and 83 SM) were used for the downstream analysis.

### High-throughput sequencing, bioinformatics and statistical analysis

Sequencing was carried out using Ion Torrent Personal Genome Machine (PGM) with the Ion 318 Chip Kit v2 (Thermo Fisher Scientific, Waltham, Massachusetts, U.S.A) under manufacturer’s conditions. The raw sequences have been submitted to NCBI under the Bioproject accession number PRJNA384692. Raw reads or FASTA sequences were de-multiplexed, quality-filtered and analysed using Quantitative Insights Into Microbial Ecology (QIIME) 1.9.1 software [40].

As parameters for the analysis, we considered a sequence length greater than 300 bp, a mean quality score above 25 in sliding window of 50 nucleotides, no mismatches on the primer and default values in the split libraries script. VSearch (version 1.11.1) was used to dereplicate sequences, cluster them by de novo approach at 97% of similarity and detect and remove chimeras [41]. Taxonomy was assigned by the Ribosomal Database Project (RDP) classifier [42] using Greengenes database 13.8 [43] as reference, and then sequences were aligned through PyNAST method [44]. Reads were also filtered removing chloroplast and low abundance sequences (less than 0.005% of total Operational Taxonomic Units (OTUs)) [45].

The filtered OTU table was used to perform downstream analyses. Taxonomy showed the composition of OTUs for each sample or group of samples. Alpha and beta diversity, which analyse differences within and among samples, respectively, were carried out with a depth of 9300 sequences. Alpha diversity outputs were represented using two different metrics, describing how many taxa are present in the samples: observed species that considers only the richness or the total number of OTUs and Shannon index that estimates the evenness or the relative abundance of OTUs in addition to the richness. As the definition of subclinical mastitis is not homogeneous, an alternative classification of non-mastitic samples was carried out for the purpose of statistical analyses of alpha diversity, using four different grouping based on SCC, independently from the microbiological culture, namely: a total of 22 samples derived from clinically healthy quarters with a SCC of less than 100,000 cells/ml (class 1); 33 samples derived from clinically healthy quarters with a SCC ranging from 100,000 to 499,000 cells/ml (class 2); 14 samples derived from clinically healthy quarters with a SCC ranging from 500,000 to 100,000,000 cells/ml (class 3); 40 samples derived from clinically healthy quarters with a SCC greater than 100,000,000 cells/ml (class 4). Beta diversity, which evaluates how many taxa are shared among samples, was calculated using weighted and unweighted UniFrac distance matrices, where quantitative and qualitative approach is respectively considered in addition to the phylogenetic analysis derived from UPGMA trees. Distance matrices were plotted using the Principal Components Analysis (PCA).

Taxonomical analysis, due to the not-normal distribution of data assessed by Shapiro-wilk test, was evaluated with the non-parametric Kruskal-Wallis method and Dunn's post-hoc multiple comparison test; Bonferroni correction was also performed.

Statistical significance of alpha diversity was assessed using the non-parametric Monte Carlo test (999 permutations).

Beta diversity statistics was performed with the non-parametric Adonis and ANOSIM methods, which reflects the ANOVA test for not normally distributed samples. Statistical significance is determined by *p*-value, R^2^ value or percentage of variation explained by the variable (for Adonis method) and R value (for ANOSIM method) where more the value is close to 1, more the dissimilarity is high.

## Results

### Diagnosis of mastitis by bacterial culture and SCC

In order to identify and classify samples for Next Generation Sequencing (NGS) characterization of microbiota, milk was collected and tested for microbiological culture. Results are presented in Table 1.

**Table 1:** Microbiological culture results: Prevalence of cultured bacteria species in each group of milk samples.

Cultured bacteria	CM	CM%	H	H%	SM	SM%	Total
**Negative**	**3**	**11.1**	**27**	**100**	**15**	**18.1**	**55**
*Trueperella pyogenes*	4	14.8	0	0	1	1.2	5
*Escherichia coli*	0	0.0	0	0	1	1.2	1
*Pseudomonas aeruginosa*	2	7.4	0	0	0	0.0	2
*Streptococcus agalactiae*	1	3.7	0	0	1	1.2	2
*Staphylococcus aureus*	5	18.5	0	0	37	44.6	42
*Staphylococcus aureus-Streptococcus agalactiae*	3	11.1	0	0	4	4.8	7
*Staphylococcus chromogenes*	0	0.0	0	0	2	2.4	2
*Streptococcus dysgalactiae*	4	14.8	0	0	3	3.6	7
*Staphylococcus*. spp.	0	0.0	0	0	18	21.7	18
*Staphylococcus*. spp. *-Escherichia coli*	1	3.7	0	0	0	0.0	1
Contaminated and/or missing	4	14.8	0	0	1	1.2	5
**Total**	**27**	100.0	**27**	100	**83**	100.0	**137**

Only samples used for microbiota determination were included.

All milk samples from healthy quarters had negative microbiological cultures and a SCC < 200,000 cells/ml.

Among SM affected quarters, bacteria that are potentially associated with mastitis were recovered in 67 samples (81%), whereas the others 15 were negative after microbiological culture with SCC > 200.000 cells/ml. For 1 sample, microbiological results were missing.

All the samples collected from quarters with CM contained bacteria that are associated with mastitis, as detected under standard growing conditions, except for 3 samples that were negative, and 4 samples whose microbiological results were missing (nr. 2) or contaminated (nr.2). No sample was found positive for Mycoplasma.

### Ion torrent output: Sequence results after filtering processes

The sequencing of 137 milk samples produced 31,777,423 total reads with an average read length of 217.5 nucleotides, a median of 259.5 nucleotides and a mode of 346 nucleotides. Before removing chloroplast sequences, 16,231 OTUs were found. After chloroplast, low abundance filtering and removal of two samples as previously described, 1,398 OTUs were obtained.

### Core microbiota and taxonomic profile analysis

**Fig 1:**
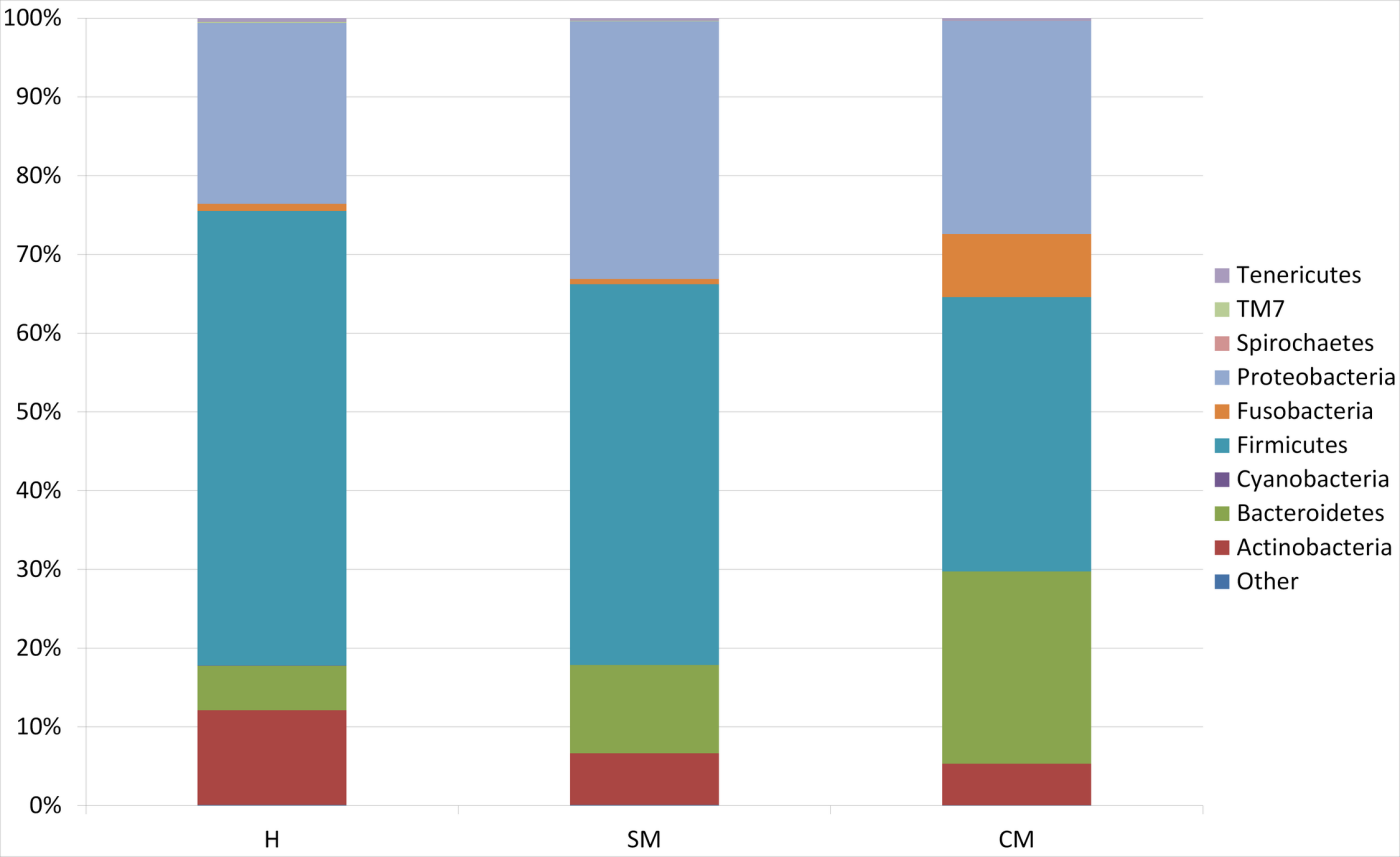
Water buffalo milk taxonomic profile at phylum level. Microbiota composition at the phylum level for the 16S rRNA. H = Healthy samples; SM = Sub-Clinical mastitis samples; CM = Clinical mastitis samples

Water buffalo milk microbiota is composed of 9 main phyla, namely *Actinobacteria*, *Bacteroidetes*, *Cyanobacteria*, *Firmicutes*, *Fusobacteria*, *Proteobacteria*, *Spirochaetes*, *TM7* and *Tenericutes* (Fig 1 and Table 2).

**Table 2:** Relative abundance of microbiota taxa at phylum level.

	*Relative abundance frequences*			*p-value (where p<0*.*05)*		
	H	SM	CM	H vs SM	H vs CM	SM vs CM
Other	0.08%	0.11%	0.06%	ns	ns	Ns
*Actinobacteria*	12.04%	6.55%	5.26%	0.043	0.053	Ns
*Bacteroidetes*	5.66%	11.22%	24.44%	ns	ns	Ns
*Cyanobacteria*	0.03%	0.00%	0.00%	ns	ns	Ns
*Firmicutes*	57.70%	48.33%	34.83%	ns	ns	Ns
*Fusobacteria*	0.94%	0.66%	8.00%	ns	0.001	<0.0001*
*Proteobacteria*	22.93%	32.71%	27.11%	ns	ns	Ns
*Spirochaetes*	0.01%	0.02%	0.02%	ns	ns	Ns
*TM7*	0.14%	0.05%	0.02%	0.039	0.004	Ns
*Tenericutes*	0.47%	0.35%	0.25%	ns	0.011	Ns

H = Healthy samples; SM = Sub-Clinical Mastitis samples; CM = Clinical mastitis samples. Significance at ≤ 0.05.

* Bonferroni correction was applied.

The healthy milk microbiota is dominated by *Firmicutes*, representing the 57.70% of the bacteria, followed by *Proteobacteria* (23%), *Actinobacteria* (12%), *Bacteroidetes* (6%) and *Fusobacteria* (1%).

As compared to milk from H animals, SM milk presents a decrease of *Firmicutes (48%) and Actinobacteria (6%)* and a relative increase in *Bacteroidetes* (11%) and *Proteobacteria (33%)*. In CM milk, the relative abundance of *Bacteroidetes* increases to 24% and *Fusobacteri*a to 8%, whereas *Proteobacteria*, *Tenericutes* and *Actinobacteria* were decreased. Statistical differences are presented in Table 2. Only *Fusobacteria* phylum was found to be statistically significantly different between SM and CM samples. Results were also analysed at family level: relative abundances and statistical differences (*p* ≤ 0.05) are presented in S1 Fig and S1 Table, considering the main families (relative frequency at least at 1%). *Peptostreptococcaceae*, *Aerococcaceae*, *Staphylococcaceae*, *Clostridiaceae*, *Moraxellaceae* and *Corynebacteriaceae* accounted for 69% of the families of H milk. Among the major families (> 8%), *Peptostreptococcaceae*, *Aerococcaceae* and *Staphylococcaceae* decreased in a statistically significant way in SM milk. Together with *Staphylococcaceae* and *Moraxellaceae*, *Aerococcaceae*, *Clostridiaceae*, *Corynebacteriaceae* and *Peptostreptococcaceae* are decreased in CM milk as well. As compared with SM milk, CM milk presented an increase of *Porphyromonadaceae*, *Fusobacteriaceae* and *Leptotrichiaceae*, and a decrease of *Staphylococcaceae* and *Moraxellaceae*.

**Fig 2:**
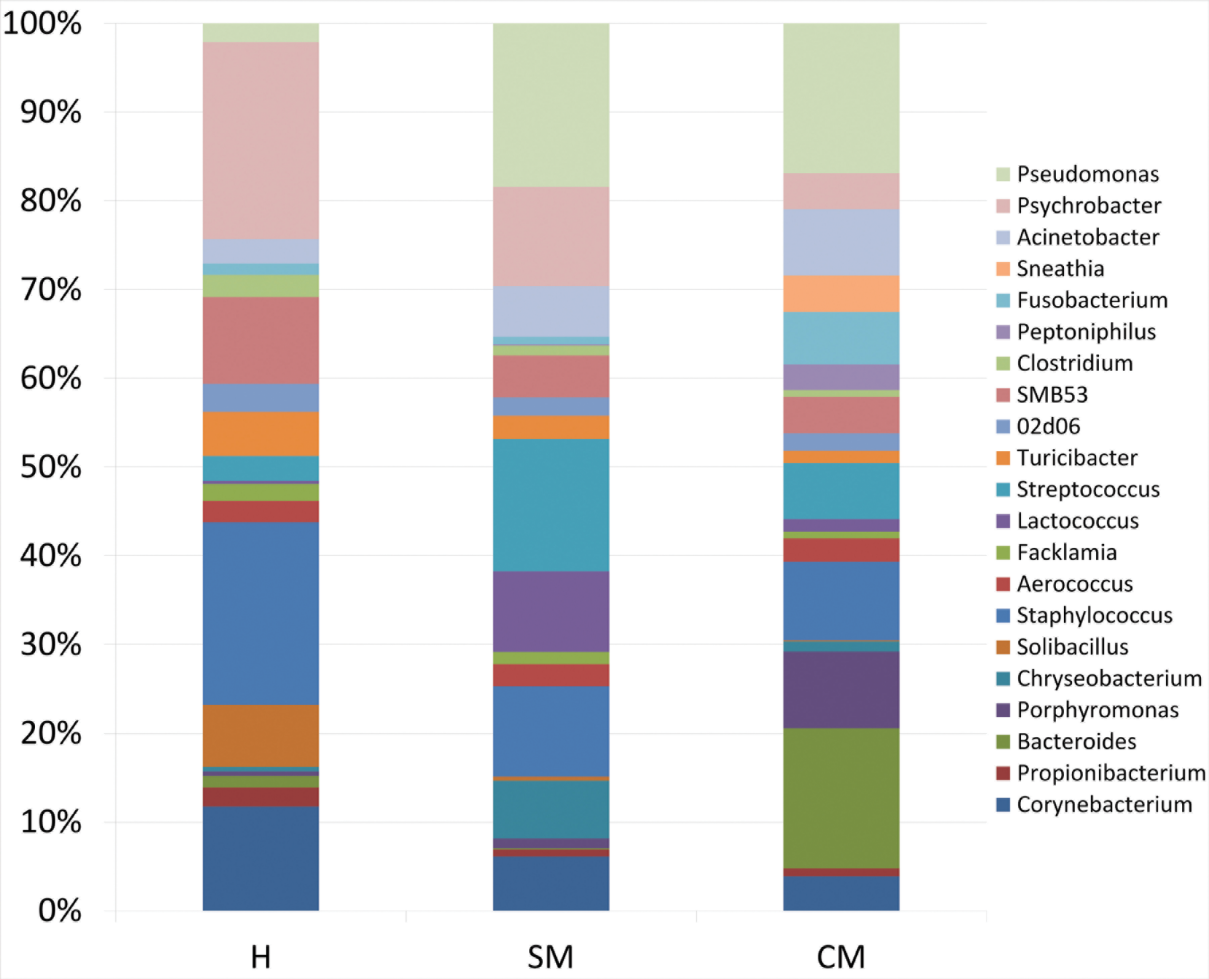
Water buffalo milk taxonomic profile at genus level. **Microbiota composition at the genus level for the 16S rRNA gene** Microbiota composition at the genus level for the 16S rRNA gene. H = Healthy samples; SM = Sub-Clinical mastitis samples; CM = Clinical mastitis samples.

The modifications at family level reflect on those at genus level (Fig 2 and Table 3) (relative frequency at least at 1%).

**Table 3:** Relative abundance frequencies at genus level. Grouping following mastitis diagnosis.

	*Relative abundance frequencies*			*p-value (where p<0*.*05)*		
Genera	H	SM	CM	H vs SM	H vs CM	SM vs CM
*Corynebacterium*	8.61%	4.80%	3.10%	ns	0.012	ns
*Propionibacterium*	1.57%	0.61%	0.71%	0.002	0.004	ns
*Bacteroides*	0.95%	0.11%	12.60%	ns	0.018	<0.001
*Porphyromonas*	0.37%	0.86%	6.88%	0.051	<0.0001*	<0.0001*
*Chryseobacterium*	0.39%	5.12%	0.92%	ns	ns	0.016
*Solibacillus*	5.12%	0.37%	0.10%	<0.0001	<0.0001*	0.025
*Staphylococcus*	15.09%	7.98%	7.05%	ns	0.017	0.005
*Aerococcus*	1.76%	1.97%	2.10%	ns	0.007	0.006
*Facklamia*	1.41%	1.08%	0.60%	0.053	<0.001	0.018
*Lactococcus*	0.25%	7.11%	1.13%	ns	ns	ns
*Streptococcus*	2.04%	11.70%	5.04%	ns	ns	ns
*Turicibacter*	3.66%	2.06%	1.11%	ns	<0.0001	0.001
*02d06*	2.31%	1.63%	1.56%	ns	<0.0001	0.002
*SMB53*	7.18%	3.70%	3.28%	0.018	0.001	ns
*Clostridium*	1.82%	0.88%	0.63%	0.009	<0.0001	<0.001
*Peptoniphilus*	0.00%	0.10%	2.28%	ns	<0.0001	<0.001
*Fusobacterium*	0.94%	0.66%	4.74%	ns	<0.0001	<0.0001*
*Sneathia*	0.00%	0.00%	3.26%	ns	0.007	<0.0001
*Acinetobacter*	2.03%	4.47%	5.97%	ns	ns	ns
*Psychrobacter*	16.26%	8.79%	3.22%	ns	0.002	0.027
*Pseudomonas*	1.57%	14.45%	13.48%	ns	ns	ns
*Micrococcus*	0.53%	0.14%	0.21%	<0.0001	<0.001	NS
*Flavobacterium*	0.06%	0.51%	0.49%	ns	Ns	ns
*Jeotgalicoccus*	0.82%	0.46%	0.26%	<0.001	<0.0001	ns
*Trichococcus*	0.82%	0.74%	0.48%	0.03	<0.001	0.007
*Helcococcus*	0.16%	0.21%	1.84%	ns	ns	0.014
*Roseomonas*	0.02%	0.55%	0.00%	ns	ns	ns
*Erwinia*	0.01%	0.51%	0.00%	ns	ns	0.016

*Bonferroni-corrected p-value at 0.0001

**Fig 3:**
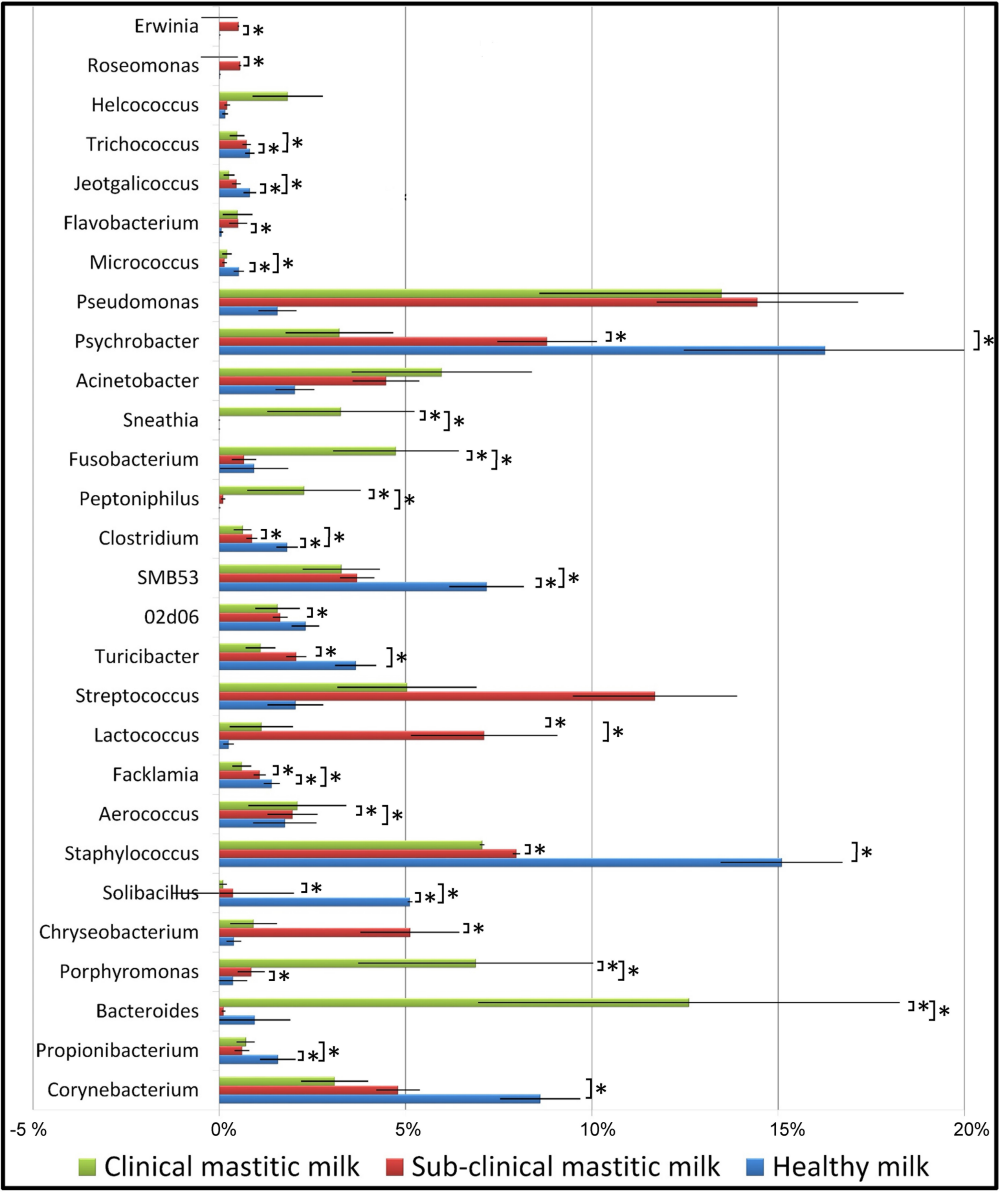
Water buffalo milk taxonomic profile at genus level. The microbial relative abundance at genus level between: H = Healthy samples; SM = Sub-Clinical mastitis samples; CM = Clinical mastitis samples; * indicates statistical significance (*p* ≤ 0.05).

**Fig 4:**
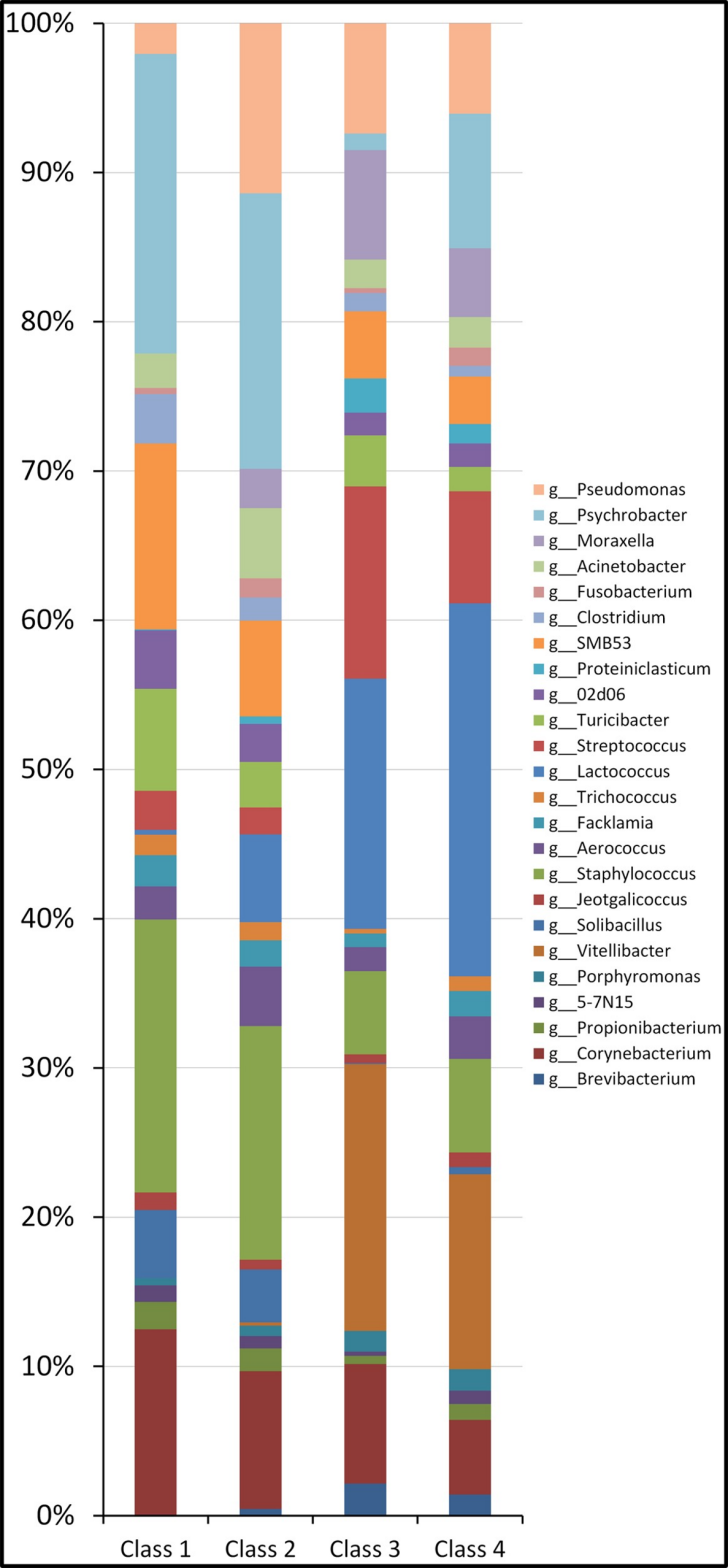
Water buffalo milk microbiota composition at the genus level for the 16S rRNA gene after classification of clinically healthy samples in SCC classes. Microbiota composition at the genus level for the 16S rRNA gene: Class 1, with a SCC < 100,000, Class 2, with a SCC between 100,000 and 500,000, Class 3, with a SCC between 500,000 and 1,000,000 and Class 4, with a SCC > 100,000,000.

The water buffalo core microbiota at genus level, defined as the asset of genera shared by all healthy milk samples, included 15 genera, namely *Micrococcus*, *Propionibacterium*, *5-7N15*, *Solibacillus*, *Staphylococcus*, *Aerococcus*, *Facklamia*, *Trichococcus*, *Turicibacter*, *02d06*, *SMB53*, *Clostridium*, *Acinetobacter*, *Psychrobacter and Pseudomonas*. As compared to H quarters, milk from SM presents a statistically significant decrease of *Propionibacterium*, *Solibacillus*, SMB53, and *Clostridium*, and an increase of *Porphyromonas*. Milk obtained from CM evidenced a further decrease of most of the genera found with a relative abundance more than 1%, and an increase of *Bacteroides*, *Porphyromonas*, *Aerococcus*, *Lactococcus*, *Peptoniphilus*, *Fusobacterium*, *Sneathia* and *SM853*. As compared to SM, CM milk samples present a decrease of *Staphylococcus*, *Turicibacte*r, *02d06*, *SMB53*, *Clostridum* and *Psychrobacter*, and an increase of *Bacteroides*, *Porphyromonas*, *Aerococcus*, *Peptoniphilus*, *Fusobacterium* and *Sneathia*. Fig 3 presents the microbial relative abundance at genus level in H, SM and CM milk samples. A classification of samples independent on microbiology and based on SCC was also carried out. The samples were grouped in four SCC classes: Class 1, with a SCC < 100,000 cells/ml, Class 2, with a SCC between 100,000 cells/ml and 500,000 cells/ml, Class 3, with a SCC between 500,000 cells/ml and 1,000,000 cells/ml and Class 4, with a SCC > 1,100,000 cells/ml. Results of relative abundance of genera are reported in Fig 4 and Table 4.

**Table 4:** Relative abundance frequencies at genus level. Grouping following SCC classes.

	*Relative abundance—SCC group*				*p-values*					
*Genus/Classes*	Class 1	Class 2	Class 3	Class 4	1 vs 2	1 vs 3	1 vs 4	2 vs 3	2 vs 4	3 vs 4
*Brevibacterium*	0.02%	0.32%	0.92%	0.81%	ns	ns	ns	ns	ns	ns
*Corynebacterium*	8.97%	6.51%	3.45%	2.87%	ns	ns	0.036	ns	0.046	ns
*Propionibacterium*	1.33%	1.08%	0.23%	0.62%	ns	0.012	0.002	ns	ns	ns
*5-7N15*	0.80%	0.59%	0.12%	0.51%	ns	0.016	0.002	ns	0.029	ns
*Porphyromonas*	0.35%	0.48%	0.60%	0.82%	ns	ns	ns	ns	ns	ns
*Vitellibacter*	0.00%	0.16%	7.69%	7.49%	ns	ns	ns	ns	ns	ns
*Solibacillus*	3.29%	2.50%	0.03%	0.28%	ns	0.001	<0.0001	0.036	0.001	ns
*Jeotgalicoccus*	0.85%	0.46%	0.26%	0.56%	ns	<0.0001	<0.0001	0.006	0.009	ns
*Staphylococcus*	13.18%	11.04%	2.39%	3.61%	ns	ns	ns	ns	ns	ns
*Aerococcus*	1.60%	2.82%	0.70%	1.63%	ns	ns	ns	ns	0.05	ns
*Facklamia*	1.51%	1.23%	0.39%	0.98%	ns	ns	ns	ns	ns	ns
*Trichococcus*	0.98%	0.87%	0.13%	0.55%	ns	ns	0.027	ns	ns	ns
*Lactococcus*	0.24%	4.14%	7.21%	14.35%	ns	0.008	ns	ns	ns	0.009
*Streptococcus*	1.88%	1.28%	5.54%	4.31%	ns	ns	ns	ns	ns	ns
*Turicibacter*	4.92%	2.14%	1.47%	0.94%	ns	ns	0.039	ns	ns	ns
*02d06*	2.83%	1.80%	0.65%	0.90%	ns	ns	0.029	ns	ns	ns
*Proteiniclasticum*	0.04%	0.35%	0.98%	0.75%	ns	ns	ns	ns	0.05	ns
*SMB53*	8.98%	4.53%	1.94%	1.82%	ns	ns	0.004	ns	0.031	ns
*Clostridium*	2.38%	1.09%	0.53%	0.41%	ns	0.014	0.001	ns	0.018	ns
*Fusobacterium*	0.30%	0.89%	0.14%	0.70%	ns	ns	ns	ns	ns	ns
*Acinetobacter*	1.66%	3.32%	0.82%	1.17%	ns	ns	ns	ns	ns	ns
*Moraxella*	0.00%	1.87%	3.15%	2.66%	ns	ns	ns	ns	ns	ns
*Psychrobacter*	14.47%	13.01%	0.48%	5.16%	ns	ns	ns	ns	ns	ns
*Pseudomonas*	1.48%	8.03%	3.18%	3.48%	ns	ns	ns	ns	ns	ns

Class 1: SCC < 100,000 cells/ml; class 2: SCC between 100,000 cells/ml and 499,000 cells/ml; class 3: SCC between 500,000 cells/ml and 1,000,000 cells/ml; class 4: with a SCC > 1,000,000 cells/ml.

No “core microbiota” could be defined following a classification of samples in SCC classes. As compared to SCC class 1, the relative abundance of *Jeotgalicoccus* was decreased from 0.85% of Class 1 to 0.56% of class 4. The relative abundance of *Corynebacterium*, *Solibacillus*, *SMB53* and *Clostridium* was decreased as well from class 1 to class 4. On the contrary, the relative abundance of *Lactococcus* was increased, from 0.24% of class 1 to 14.35% of class 4, although it must be said that only differences between class 1 and class 3, and 3 to class 4 were statistically significant.

### Discriminant analysis and clustering of samples

**Fig 5:**
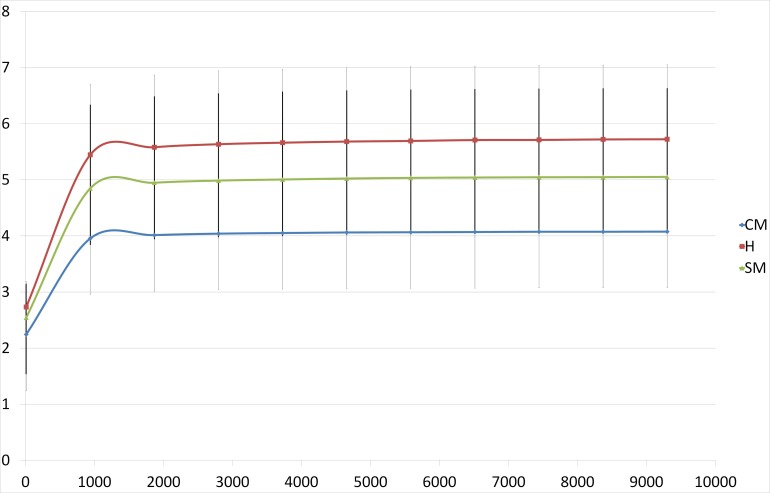
Alpha diversity analysis. Rarefaction curves of samples with regards to quarter patho-physiological status (CM: clinical mastitis; H: healthy; SM: sub-clinical mastitis), as defined by the Shannon index. Statistical difference is present between H and CM groups (*p* = 0.03).

Alpha diversity analysis showed that H and CM samples were statistically different with 445.76 (STD = ± 140.82) and 198.89 (STD = ± 186.28) observed OTUs (*p* = 0.006) and 5.72 (STD = ± 1.33) and 4.08 (STD = ± 2.05) Shannon index (*p* = 0.03), respectively. Statistical differences were also found comparing H with SM group (*p* = 0.018) with 445.76 and 317.15 (STD = ± 178.70) observed OTUs, respectively. Alpha diversity is plotted in Fig 5 using Shannon index. On the contrary, it is not possible to discriminate between SM and CM samples. As the definition of subclinical mastitis is not homogeneous, an alternative classification of non-mastitic samples was carried out, using parameters that are independent from microbiological results, alternatively classifying the healthy and sub-clinical mastitis samples in four different grouping based on SCC. Results are presented in S2 Fig. Class 1 (SCC < 100,000 cells/ml) and 4 (SCC > 1,000,000 cells/ml) were statistically different with 468.19 (STD = ± 126.31) and 266.93 (STD = ± 159.87) observed OTUs (*p* = 0.006) and 6.61 (STD = ± 1.11) and 4.91 (STD = ± 1.6) Shannon index (*p* = 0.006), respectively. Also class 1 and 3 (SCC between 500,000 and 1,000,000 cells/ml) were statistically different, with 6.61 (STD = ± 1.11) and 4.29 (STD = ± 1.3) Shannon index (*p* = 0.006), respectively. Class 2 (SCC between 100,000 and 499,000 cells/ml) and 4 were statistically different for observed OTUs, 391.15 (STD = ± 166.03) and 266.93 (STD = ± 159.87), respectively (*p* = 0.03).

**Fig 6:**
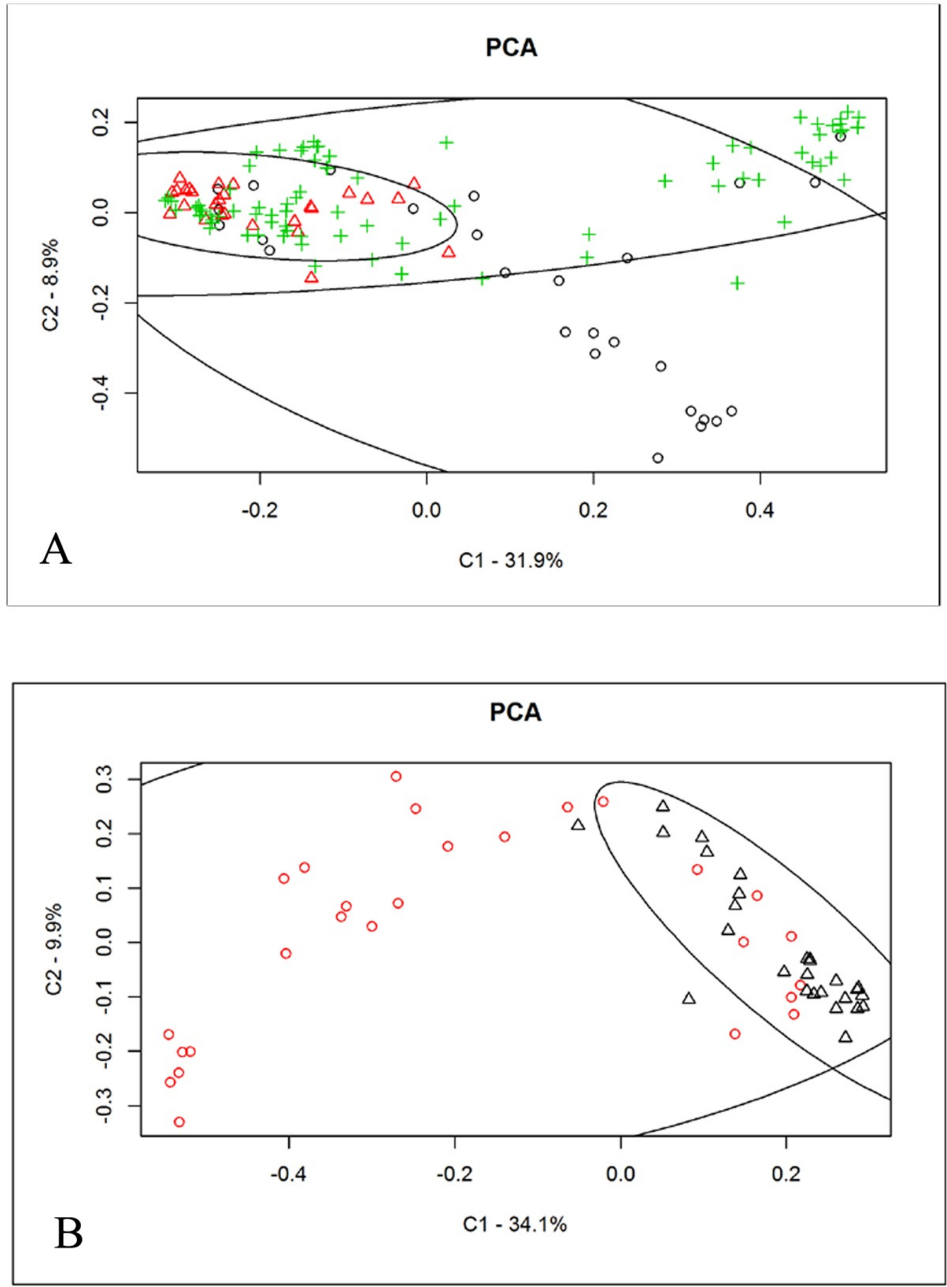
Beta diversity analysis. Unweighted Unifrac analysis including H (Healthy) and CM (Clinical mastitis) samples. Adonis: R = 0.17 p = 0.001 ANOSIM: R = 0.37 *p* = 0.001. Panel A: results including H, SM and CM quarters. Panel B: results including only H and CM. o = CM; + = SM; Δ = H

Beta diversity analysis was carried out comparing milk samples from healthy and clinical and sub-clinical quarters, using the weighted and unweighted Unifrac distance metric. The results provided the evidence that it is possible to discriminate between the groups (Adonis: R^2^ = 0.09 and p = 0.001; ANOSIM: R = 0.15 and p = 0.003 for weighted Unifrac; Adonis: R^2^ = 0.09 and p = 0.001; ANOSIM: R = 0.09 and p = 0.0012 for unweighted Unifrac). Beta diversity is plotted in using unweighted Unifrac Fig 6 (Panel A): the first component (C1) explains the 31.9% of the variability and separate healthy from clinical mastitis milk samples, even if some overlaps are present. On the other hand, the second component (C2) explains the 8.9% and separates clinical mastitis samples from the others, although with some overlaps. Considering only H and CM groups, where C1 = 34.1% and C2 = 9.9%, the separation of healthy and clinical mastitis samples is improved as shown in Fig 6 (Panel B) (Adonis: R^2^ = 0.09 and p = 0.001; ANOSIM: R = 0.15 and p = 0.003 for weighted Unifrac; Adonis: R^2^ = 0.17 and *p* = 0.001; ANOSIM: R = 0.37 and *p* = 0.001 for unweighted Unifrac), showing that H quarters can be discriminated from CM quarters by C1. Box Plot representing C1 and C2 axes are presented in S3 Fig. Beta diversity analysis was also carried out comparing the four SCC groups derived from all clinically healthy quarters, using the weighted and unweighted Unifrac distance metric (Adonis: R^2^ = 0.08 and p = 0.001; ANOSIM: R = 0.09 and p = 0.003 for weighted Unifrac; Adonis: R^2^ = 0.08 and p = 0.001; ANOSIM: R = 0.06 and p = 0.017 for unweighted Unifrac). Results are presented in S4 Fig and show that the distribution of class 3 (SCC between 500,000 and 1,000,000 cells/ml) and 4 (SCC > 1,000,000 cells/ml) was more scattered in the plot compared to class 1 (SCC < 100,000 cells/ml) and 2 (SCC between 100,000 and 499,000 cells/ml), which were more homogeneous, and better clusterized by C1 (component one) axis that explains the 36.4% of the variability. C2 (component two) axis cannot discriminate between groups. Box Plot representing C1 and C2 axes are presented in S4 Fig.

## Discussion

We report here the first detailed characterization of milk microbiota in water buffaloes with clinical and sub-clinical mastitis as determined by 16S rRNA gene diversity profiling. Therefore, being the ribosomal 16S RNA gene domain restricted to bacteria and archaea [46] we did not address the eukaryote content of milk. The microbiota of milk from healthy quarters was determined as well, providing the evidence that the OTU diversity of milk from healthy quarters is much wider that samples with clinical and sub-clinical mastitis consistently with what has been already reported in bovine milk [16, 19], colostrum [47] and teat microbiota [48]. Discriminant analysis models of water buffalo milk showed that samples collected from healthy quarters can be discriminated from samples derived from clinical and sub-clinical mastitis, in agreement with what was observed in bovine milk [15, 19]. On the contrary it was not possible to discriminate in clusters samples derived from SM quarters. The clustering of H milk samples was improved removing SM quarters, which were more scattered in the plot; in fact, sub-clinical samples might share healthy or clinical mastitis features such as absence of inflammatory reaction or positive bacterial culture, respectively.

The water buffalo health milk core microbiota, i.e. the number and the identity of genera that are shared among different individuals, contained 15 genera, of which *Staphylococcus* and *Psycrobacter* were the most prevalent.

The microbiota from water buffalo healthy milk is different as compared with that of bovine milk, where the core microbiota includes *Faecalibacterium*, unclassified *Lachnospiraceae*, *Propionibacterium* and *Aeribacillus* [15], and human milk, where nine genera, namely *Staphylococcus*, *Streptococcus*, *Serratia*, *Pseudomonas*, *Corynebacterium*, *Ralstonia*, *Propionibacterium*, *Sphingomonas*, *Bradyrhizobiaceae*, were present [49].

Together with *Streptococcus*, which ranges from 2% in H samples to 11.7% in SM samples, *Staphylococcus* genus was already reported as being part of core microbiota of human [49] and bovine milk [15, 19, 50, 51]. Although found in all healthy milk samples, *Pseudomonas* genus relative abundance in water buffalo milk was limited (1.5%) as compared to bovine non-mastitic milk (18.75%) [19].

The finding of *Psychrobacter* has already been reported in milk from dairy cows [19, 52], although in cow’s healthy milk the average relative abundance of *Psychrobacter* is limited (4.9%) as compared to what found in water buffalo milk (16.26%). The relative abundance of *Psychrobacter* in water buffalo milk is related to the healthy status of the mammary gland, decreasing to 3.2% in SM milk, and absent in 22% of the CM milk samples. No species belonging to *Psychrobacter* has been so far associated to mastitis. Cold-adapted *Psychrobacter* genus has been recently related to anti-biofilm activities against *Staphylococci* and *Pseudomonas aeruginosa* bacteria [53]. This finding is remarkable, since provides clues to potentiate non-antibiotic relying resistance against mammary gland pathogens. Of the other most prevalent genera that were found in all the H samples, *SMB53* and *Solibacillus* were present with the highest relative abundance, 7.17% and 5.11%, respectively. This is the first time that these two genera were found in milk. *SMB53* belongs to the family of *Clostridiaceae* and was found within the ileal bacterial community in grazing goats [54]. *Solibacillus* genus was identified among faecal bacterial community in dairy cows during subacute ruminal acidosis [55], but its presence in milk is reported here for the first time. Both *SMB53* and *Solibacillus* are regarded as faecal contaminants. On the background that all the farms included in the present study were equipped with bathing pools, that are of paramount importance for water buffaloes, in order to mitigate thermal stress, we may hypothesize that, due to the immersion of the teats in water, faecal contaminants were included in the microbiota of healthy water buffalo mammary gland. Furthermore, a recently published investigation highlighted differences between samples obtained directly from the udder cistern using a needle and vacuum and samples collected conventionally [56]. The authors suggested that contamination from teat skin, or environmental sources, may occur, interfering with the microbiological analysis and PCR-based bacteriological results. It could not be ruled out that contamination from skin and environmental sources could have occurred, affecting the taxonomic composition. It must also be said that both *Solibacillus* and *SMB53* decreased in a statistically significant way in CM milk, and therefore it is unlikely that their presence in healthy milk is related to contamination during collection of samples.

In milk from sub-clinical affected quarters, two genera, namely *Acinetobacter* and *Pseudomonas*, with a relative abundance of 4.47% and 15.09%, respectively, were present in all the samples. The presence of *Acinetobacter* was previously found in a cultured-independent study on the teat apex [57]. The involvement of *Acinetobacter* in the development of mastitis is unfrequent [58]. The presence of *Pseudomonas* was already reported in water buffalo milk [35, 59]. *Pseudomonas* is a known agent of mastitis pathogen in ruminants including cow [60], sheep [61] and goats [62], but little information is available in water buffalo species. The relative abundance of *Pseudomonas* genus was found as prevalent (18.75%) in milk from healthy cows, and decreased to 3.84% in clinical mastitis [19]. In the present study the relative abundance of *Pseudomonas* in healthy milk samples was found be limited (1.5%) as compared to SM (14.74%) and CM (13.48%) milk, but this increase was not found to be statistically significant.

No common genus was present in milk from quarters with clinical mastitis. In several samples the bacteria identified by aerobic microbiological culture corresponded to the most frequent bacterial species found after 16s rRNA gene sequencing. Moreover, many anaerobic bacterial sequences including those belonging to *Bacteroides*, *Porphyromonas* and *Fusobacterium*, were also identified. In some samples the relative abundance of these genera, such as for examples *Bacteroides* in C44 (r.a. = 92%), C56 (r.a. = 87%), C57 (r.a. = 82%), C48 (r.a. = 61%), *Porphyromonas* in C55 (r.a. = 62%) and *Fusobacterium* in C49 (r.a. = 42%), was predominant. This finding is consistent with others reported in previous studies on bovine milk [15], which detected anaerobic bacteria in both healthy and clinical mastitis affected samples, in particular those caused by *Trueperella* pyogenes. Anaerobic genera have been already found in bovine milk, although they were more frequently included in the list of gut microbes [20], and in summer mastitis [63], [64]. The presence of anaerobic genera was also found in teat microbiota as correlated to mastitis history [48]. The relative abundance of *Bacteroides* and *Porphyromonas* in healthy water buffalo is limited, and no traces of Fusobacterium sequences were found at all. In clinical mastitis samples, on the contrary, *Bacteroides*, *Fusobacterium* and *Porphyromonas* were found to be associated with mastitis where the main pathogen identified after microbiological culture was *Trueperella* (C44, C45, C57 and C58) or *Streptococcus dysgalactiae* (C49), suggesting in water buffalo as well a synergistic action between these genera, in particular where *Trueperella* is involved [64]. Discrimination between clinical and healthy quarters is significant, even if the not homogeneous microbiota profile of clinical samples needs to be deeply investigated. A different criterion to classify non clinical mastitic samples was also considered, aiming to relate microbiota profiles with inflammatory parameters, such as SCC. Therefore, on this background, samples were classified in four classes depending on SCC. Following this classification, considering alpha-diversity, and independently on microbiological culture, did not allow to cluster samples, since an overlapping between class 1 with class 2, class 2 with class 3 and class 3 with class 4 were demonstrated. This results confirm the hypothesis that classifications of water buffalo mastitis following SCC need further investigations, as previously suggested [31].

## Conclusion

The present study investigated the milk water buffalo microbiota from healthy quarters and sub-clinical and clinical mastitis, following a culture-independent metagenome approach, providing a first step in the evaluation of the microbial population in water buffalo milk, and contributing to identify the core microbiota in healthy milk. Our findings revealed the presence of genera that could not be assessed by culture-based analysis, such as *Psychrobacter*, SMB53 and *Solibacillus* whose relative decrease was associated with clinical mastitis. Open questions remain to be answered, including the relationship between microbiota with parity and different stages of lactation, as well as the relationship between farming conditions and microbiota.

## Supporting information

S1 Fig Water buffalo milk taxonomic profile at family level. Microbiota composition at the family level for the 16S rRNA gene. H = Healthy samples; SM = Sub-Clinical mastitis samples; CM = Clinical mastitis samples
(TIF)

S2 Fig Alpha diversity analysis after classification of clinically healthy samples following SCC grouping. Rarefaction curves of the four SCC groups (Class 1, with a SCC < 100,000, Class 2, with a SCC between 100,000 and 500,000, Class 3, with a SCC between 500,000 and 1,000,000 and Class 4, with a SCC > 100,000,000), as defined by the Shannon index.
Statistical differences are present between class 1 and (*p* = 0.006) and between class 1 and 3 (*p* = 0.006).
(TIF)

S3 Fig Beta diversity analysis presented as box plot. Panel A and B presents the C1 and C2 boxplots derived from Fig 5, Panel A, including H, SM and CM quarters. Panel C and C presents the C1 and C2 derived from Fig 5, Panel B, including H, and CM quarters.
(TIF)

S4 Fig Beta diversity analysis after classification of clinically healthy samples following SCC grouping. Unweighted Unifrac analysis including SCC groups derived from all clinically healthy quarters: class 1 with SCC of less than 100,000 cells/ml; class 2 with SCC ranging from 100,000 to 499,000 cells/ml; class 3 with SCC ranging from 500,000 to 100,000,000 cells/ml; class 4 with SCC greater than 100,000,000 cells/ml. Adonis: R^2^ = 0.08 and p = 0.001; ANOSIM: R = 0.06 and p = 0.017.
Panel A: beta diversity plot. Panel B: C1 and C2 boxplots derived from Panel A.
O = group 1; Δ = group 2; + = group 3; x = group 4.
(TIF)

S1 Table Relative abundance of microbiota taxa at family level. H = Healthy samples; SM = Sub-Clinical Mastitis samples; CM = Clinical mastitis samples.
* Bonferroni correction was applied.
(DOCX)
