# Supplementary figures and images for: The microbiota of water buffalo milk during mastitis
Source: PLoS One. 2017 Sep 19;12(9):e0184710. doi: 10.1371/journal.pone.0184710 (PMC5604978; doi:10.1371/journal.pone.0184710)

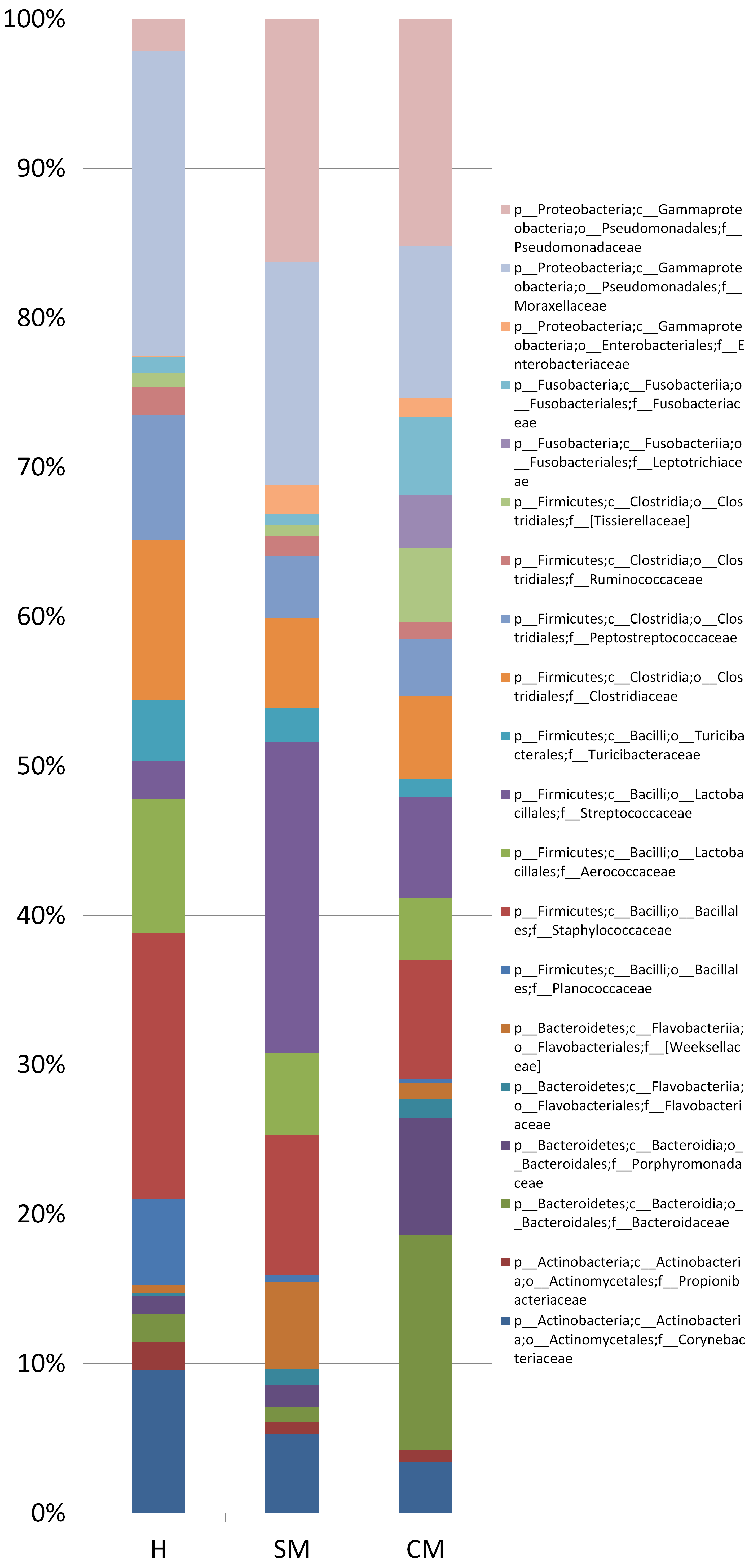

Supplement: S1 Fig — Microbiota composition at the family level for the 16S rRNA gene. H = Healthy samples; SM = Sub-Clinical mastitis samples; CM = Clinical mastitis samples (TIF) [file pone.0184710.s001.tif]

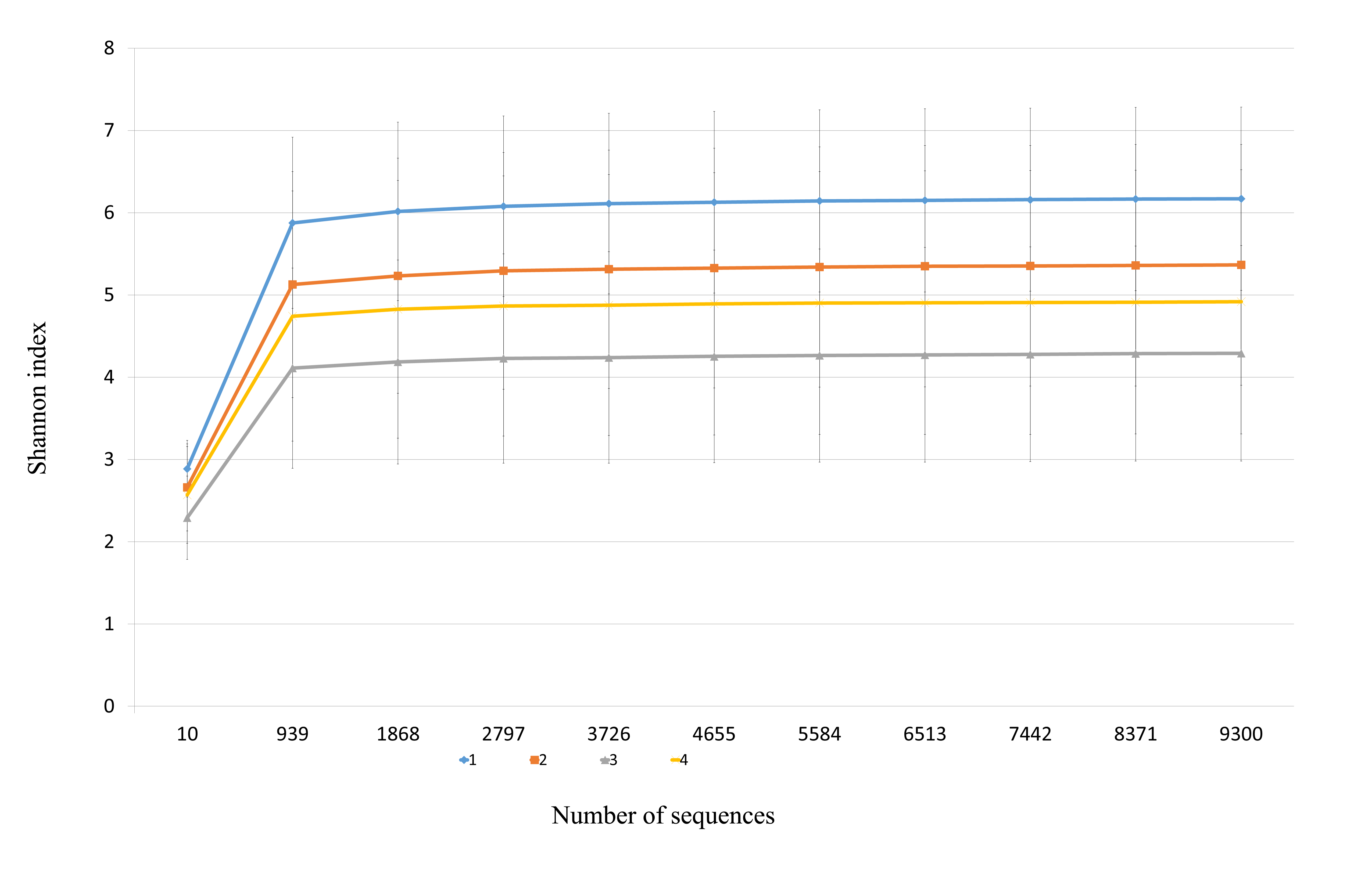

Supplement: S2 Fig — Rarefaction curves of the four SCC groups (Class 1, with a SCC < 100,000, Class 2, with a SCC between 100,000 and 500,000, Class 3, with a SCC between 500,000 and 1,000,000 and Class 4, with a SCC > 100,000,000), as defined by the Shannon index. Statistical differences are present between class 1 and (p = 0.006) and between class 1 and 3 (p = 0.006). (TIF) [file pone.0184710.s002.tif]

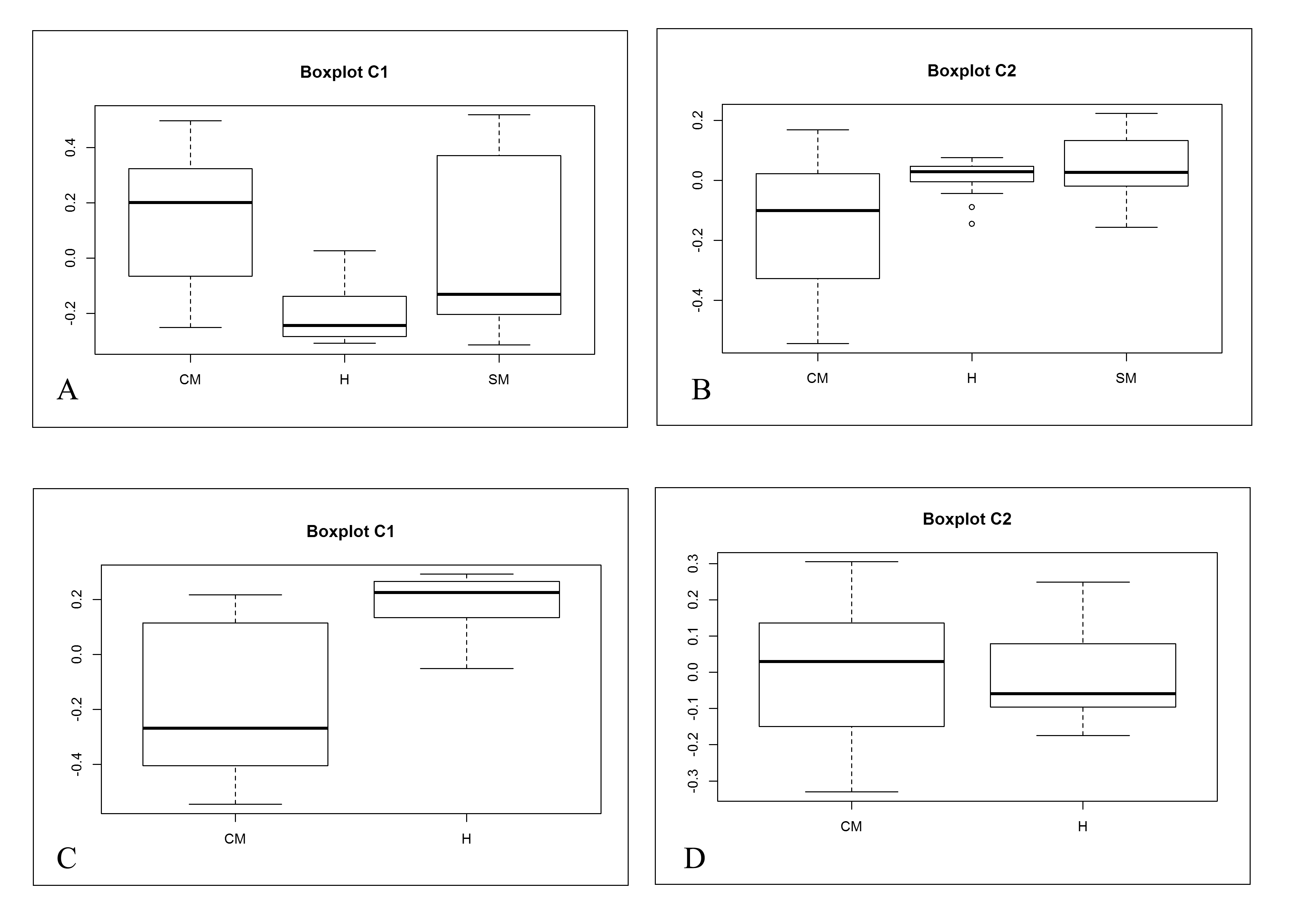

Supplement: S3 Fig — Panel A and B presents the C1 and C2 boxplots derived from Fig 5, Panel A, including H, SM and CM quarters. Panel C and C presents the C1 and C2 derived from Fig 5, Panel B, including H, and CM quarters. (TIF) [file pone.0184710.s003.tif]

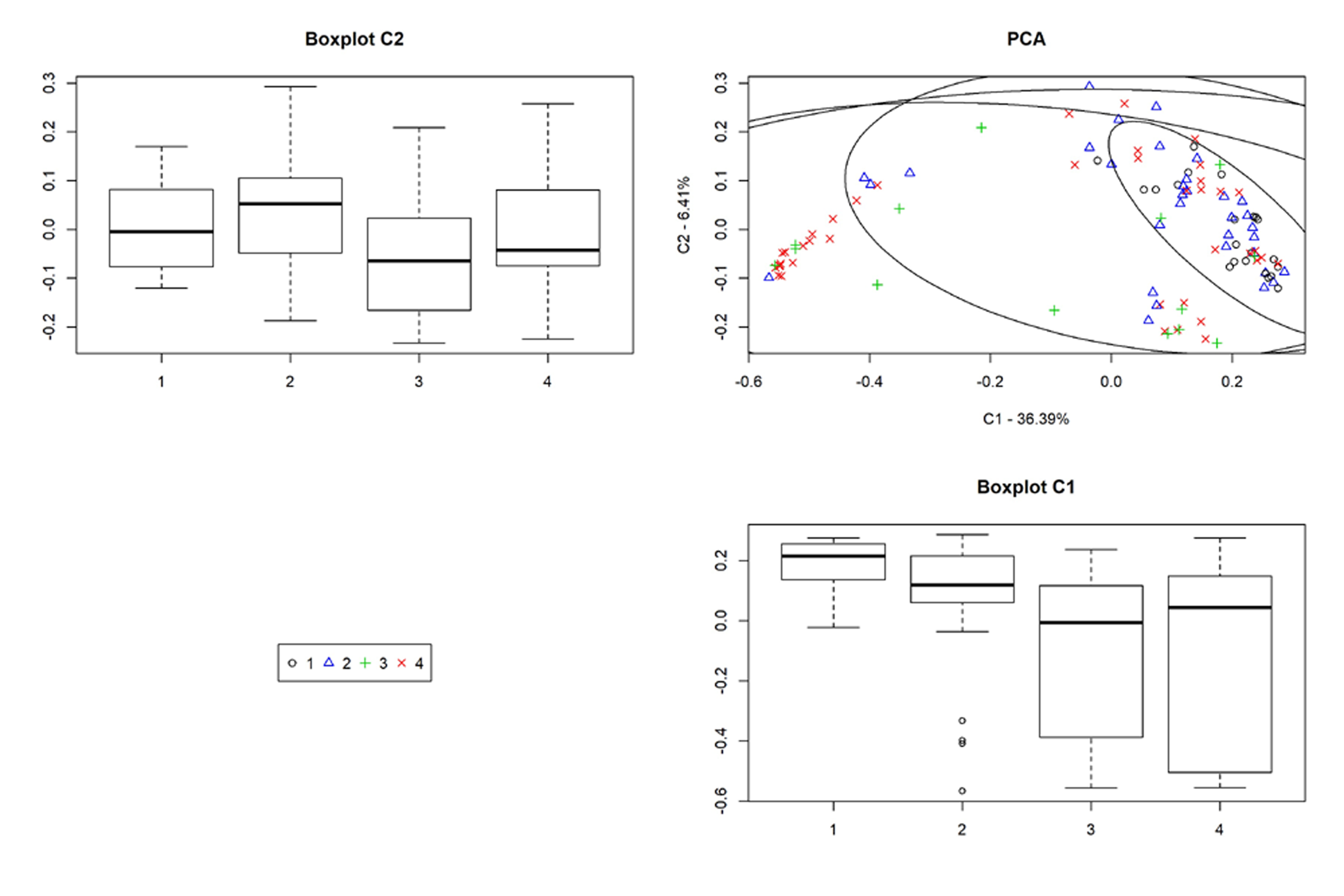

Supplement: S4 Fig — Unweighted Unifrac analysis including SCC groups derived from all clinically healthy quarters: class 1 with SCC of less than 100,000 cells/ml; class 2 with SCC ranging from 100,000 to 499,000 cells/ml; class 3 with SCC ranging from 500,000 to 100,000,000 cells/ml; class 4 with SCC greater than 100,000,000 cells/ml. Adonis: R2 = 0.08 and p = 0.001; ANOSIM: R = 0.06 and p = 0.017. Panel A: beta diversity plot. Panel B: C1 and C2 boxplots derived from Panel A. O = group 1; Δ = group 2; + = group 3; x = group 4. (TIF) [file pone.0184710.s004.tif]
