# Supplementary material for: The microbiota of water buffalo milk during mastitis
Source: PLoS One. 2017 Sep 19;12(9):e0184710. doi: 10.1371/journal.pone.0184710 (PMC5604978; doi:10.1371/journal.pone.0184710)
Supplement: S1 Table — H = Healthy samples; SM = Sub-Clinical Mastitis samples; CM = Clinical mastitis samples. * Bonferroni correction was applied. (DOCX) [file pone.0184710.s005.docx]

**Table S1: Relative abundance of microbiota taxa at family level.**

H = Healthy samples; SM = Sub-Clinical Mastitis samples; CM = Clinical mastitis samples

* Bonferroni correction was applied

|  | *Relative abundance frequencies* | | | *p-value (where p<0.05)* | | |
| --- | --- | --- | --- | --- | --- | --- |
|  | **H** | **SM** | **CM** | **H vs SM** | **H vs CM** | **SM vs CM** |
| *Actinomycetaceae* | 1% | 0% | 0% | <0.001 | 0,005 | ns |
| *Corynebacteriaceae* | 9% | 5% | 3% | 0,005 | ns | ns |
| *Microbacteriaceae* | 0% | 0% | 1% | ns | ns | ns |
| *Micrococcaceae* | 1% | 1% | 0% | 0,05 | 0,06 | ns |
| *Propionibacteriaceae* | 2% | 1% | 1% | 0,018 | 0,004 | ns |
| *Bacteroidaceae* | 2% | 1% | 13% | 0,018 | ns | ns |
| *Porphyromonadaceae* | 1% | 1% | 7% | 0,023 | ns | 0,056 |
| *Flavobacteriaceae* | 0% | 1% | 1% | ns | ns | ns |
| *[Weeksellaceae]* | 0% | 5% | 1% | ns | ns | 0,001 |
| *Planococcaceae* | 5% | 0% | 0% | <0.0001 | <0.0001* | ns |
| *Staphylococcaceae* | 16% | 8% | 7% | ns | 0,018 | 0,022 |
| *Aerococcaceae* | 8% | 5% | 4% | 0,053 | 0,003 | ns |
| *Carnobacteriaceae* | 1% | 1% | 0% | 0,036 | <0.001 | 0,009 |
| *Streptococcaceae* | 2% | 19% | 6% | ns | ns | ns |
| *Turicibacteraceae* | 4% | 2% | 1% | 0,038 | <0.0001 | 0,001 |
| *Clostridiaceae* | 10% | 5% | 5% | 0,053 | 0,002 | ns |
| *Lachnospiraceae* | 1% | 1% | 0% | 0,011 | <0.001 | ns |
| *Peptostreptococcaceae* | 8% | 4% | 4% | 0,043 | 0,001 | 0,035 |
| *Ruminococcaceae* | 2% | 1% | 1% | 0,007 | 0,001 | ns |
| *[Tissierellaceae]* | 1% | 1% | 5% | 0,003 | ns | 0,036 |
| *Fusobacteriaceae* | 1% | 1% | 5% | ns | <0.0001 | <0.0001* |
| *Leptotrichiaceae* | 0% | 0% | 3% | ns | 0,008 | <0.001 |
| *Acetobacteraceae* | 0% | 1% | 0% | ns | ns | ns |
| *Comamonadaceae* | 0% | 0% | 1% | ns | ns | ns |
| *Oxalobacteraceae* | 0% | 0% | 1% | ns | ns | ns |
| *Enterobacteriaceae* | 0% | 2% | 1% | ns | ns | 0,031 |
| *Moraxellaceae* | 18% | 13% | 9% | ns | 0,046 | 0,041 |
| *Pseudomonadaceae* | 2% | 15% | 14% | ns | ns | ns |
